# The Personal Glucose Meter as the Measurement Principle in Point-of-Care Applications

**DOI:** 10.3390/bios15020121

**Published:** 2025-02-19

**Authors:** Mònica Cano, Manel del Valle

**Affiliations:** Sensors and Biosensors Group, Department of Chemistry, Universitat Autònoma de Barcelona (UAB), Edifici Cn, Bellaterra, 08193 Barcelona, Spain; monica.cano.garrido@gmail.com

**Keywords:** glucose biosensor, point-of-care, aptamer, antibody, invertase enzyme label

## Abstract

A personal glucose meter (PGM) is a medical device that measures blood glucose levels and can be found worldwide. Owing to their sensitivity, simplicity, portability, and low cost, PGMs stand as one of the most frequently utilized analytical methods. This work reviews the different applied methodologies for detecting analytes other than glucose employing a PGM and how it can be incorporated for point-of-care diagnosis needs. To visualize the variants, first, a classification is made according to the biorecognition elements used (aptamers, antibodies, etc.), and where the determination of different analytes is done through the glucose signal using different glucose-generating enzymes such as invertase or glucosidase. Transduction can also be based on the use of nanocarriers that generally encapsulate glucose, although it is also possible to find a combination of the two aforementioned strategies. The PGM can also be used for the direct detection of interfering substances of the biosensor, such as NADH or paracetamol. Lastly, we discuss how a PGM might have been implemented to detect COVID-19 and how it could be used on a massive scale for the point-of-care diagnosis of a pandemic.

## 1. Introduction

In 2015, the United Nations General Assembly adopted the Sustainable Development Goals as a universal call for prosperity [1]. Goal 3, health and well-being, aims to ensure healthy lives and promote well-being at all ages as an essential part of sustainable development [2,3]. Even so, a percentage of people still lack access to essential health services, including disease diagnosis and treatment [4]. Rapid diagnosis systems may help in reducing inequalities in detecting diseases, given that they do not require specific facilities or qualified personnel [5,6].

This type of rapid diagnosis is called point-of-care (POC), defined as a test that can be performed near a patient or at the site where medical care is provided. In this manner, results can be accessed immediately, as the goal of POC is to expedite medical decisions [7]. Point-of-care testing is performed using portable instruments that can be easily manipulated; therefore, no diagnostic infrastructure or qualified personnel are required. This type of testing demonstrated its utility with the recent COVID-19 pandemic, where rapid diagnostics such as antigen testing could save other dilatory laboratory tests such as PCR [8].

The development of portable sensors for the on-site detection of one or more analytes has long been pursued. A worldwide paradigm of biosensor advances is the personal glucose meter (PGM), which has enabled self-assessing glucose levels [9]. Due to their easy accessibility, speed, portability, low cost, and ease of use, together with their ubiquity, PGMs have been postulated for use in other clinical diagnostics. The operating principle is using glucose as a reporter in bioassays targeting other biomarkers [10,11,12]. Within POC analysis, the use of PGMs for the detection of different biomarkers other than glucose is an expanding innovative area. To date, bioassays for the analysis of ions, organic molecules, proteins, viruses, bacteria, etc., have been described with the involvement of different glucose transduction strategies.

The glucose sensors are classified as biosensors because the sensor incorporates a redox enzyme as the recognition element (bioreceptor). Biosensors are analytical devices formed with two basic components: a bioreceptor, such as enzymes, and a transducer, in this case, an electrode, which transforms the biochemical reaction into a measurable signal. The glucose biosensor catalyzes the oxidation of glucose; once the enzymatic reaction occurs, the electrode detects the other redox products generated, obtaining a signal proportional to the concentration of glucose in the sample analyzed [13].

In a biosensor, the recognition element can be of diverse types, such as enzymes, aptamers, antibodies, etc. [14]. When the recognition reaction happens, the appearing signal can be in the form of absorbed light, pH, mass, or charge change; this reaction is called biorecognition [15]. Glucose sensors can be classified according to the above signal on absorbance methods or fluorescence, among others, but the most common are sensors based on electrochemistry. Electrochemical PGMs based on amperometry are the most widely used due to their low cost, simplicity, and selectivity [12]. Enzymatic sensors have initially been based on the use of glucose oxidase (GOX) due to its high selectivity, among other characteristics [16]. Moreover, there is an active line of research in which enzymeless glucose sensors are sought, of special interest, for example, to enlarge stability and lifetime if used for wearable sensing devices [17].

The development of enzymatic glucose sensors is normally divided into three generations [18]. Commercial biosensors are mostly second-generation biosensors, with a glucose detection range between 0 and 600 mg/dL, depending on the transducer electrode used [19]. Second-generation glucose biosensors are based on the use of mediators during the enzymatic reaction. Mediators are small redox-active molecules that have the function of transferring the electrons of the reaction to an electrode, generating a current [16,20,21].

Glucose + GOX_(ox)_ → Gluconic Acid + GOX_(red)_ (1)

GOX_(red)_ + 2Med_(ox)_ → GOX_(ox)_ + 2Med_(red)_ + 2H^+^ (2)

2Med_(red)_ → 2Med_(ox)_ + 2e^−^ (3)

Glucose → Gluconic Acid + 2e^−^ + 2H^+^ (4)

The process can be summarized schematically in the reactions (1) to (4). Reaction (1) consists of the catalytic oxidation of glucose to gluconic acid. Next, an electron transfer occurs from the reduced GOX to the oxidized mediator; Equation (2). Finally, during the oxidation/regeneration of the mediator, an electrical signal is produced, which is used to measure glucose (Equation (3)). The global overall reaction observed is Equation (4), the catalytic-mediated oxidation of glucose.

**Figure 1:**
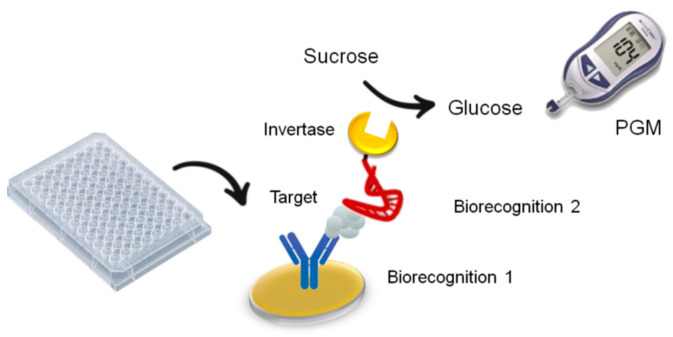
The general principle of the use of the PGM in bioassays for the determination of species other than glucose.

The first reference to the use of PGMs for detecting analytes other than glucose was proposed by Xiang and Lu in 2011 [22]. They proposed a new methodology using a conventional PGM linked with DNA aptamer sensors that detect different targets ranging from drugs such as cocaine to disease biomarkers, toxic metal ions, and even important biological cofactors such as adenosine, as sketched in Figure 1. Its operation involved generating glucose with an invertase enzyme as a label and detecting it with the PGM. The proper correlation of the target analyte and PGM signal permitted the final quantification of the former.

Some of the benefits achievable with the adoption of methodologies based on the use of PGMs in POC diagnostics are the direct and facilitated detection of biomarkers, but they can also be considered for use in food analysis, such as the detection of illegal additives or pesticides. It can also be used for environmental analysis, e.g., the detection of heavy metals [12]. The main aim of this review article is to systematically present the variants and the applicability of the use of PGMs to detect other substances. We will introduce significant applications using the PGM as a signal readout device; for this, we will present the cases in terms of the biomolecule used to extend the usability of the PGM to substances other than glucose. Namely, the biomolecules used for biorecognition have been as follows: (1) aptamers, as the first derivation in the use of PGMs; (2) antibodies; (3) peptides; (4) complementary hybridization genes; and (5) other residual cases (DNAzymes).

## 2. Type of Assays Based on the Recognition Element

### 2.1. Aptamers as the Recognition Element

Aptamers are oligonucleotides, for instance, short single-stranded DNA or RNA chains. They can recognize target molecules with high affinity and specificity by folding into a tertiary structure [23]. Their high affinity and specificity make them an effective diagnostic tool [24].

**Figure 2:**
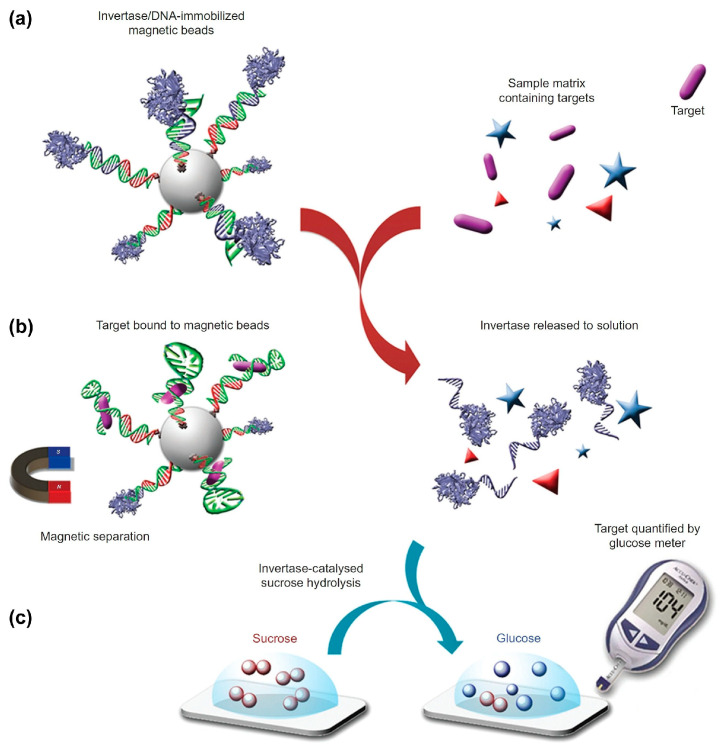
Scheme of the method using a PGM to detect a wide range of targets beyond glucose: (**a**), DNA–invertase conjugates are immobilized onto magnetic beads by DNA hybridization with a DNA aptamer that specifically responds to the target of interest. (**b**), the target interacts with the functional DNA, perturbing the DNA hybridization and causing the release of DNA–invertase conjugates to the solution. (**c**), after removal of MBs by a magnet, the DNA–invertase conjugates in solution catalyse the hydrolysis of sucrose into glucose, which is quantified by the PGM. Reprinted from Ref. [22], with permission of SpringerNature.

**Figure 3:**
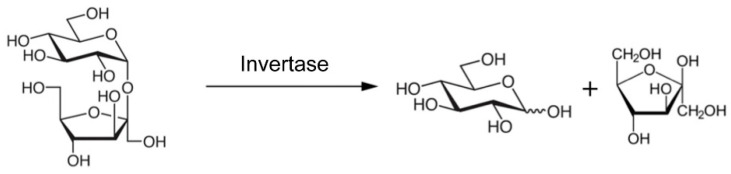
Enzymatic hydrolysis of sucrose. Source: own elaboration.

The seminal paper using the PGM to report an assay different from that of glucose and using a completely different biorecognition element was that of Xiang and Lu in 2011. In this work, they made a proof of concept with different aptamers to detect cocaine or the biomarkers adenosine and interferon-gamma of tuberculosis using the enzyme invertase as a label [22]. It was designed as a displacement assay, where the unlabeled analyte is measured for its ability to liberate a DNA-invertase conjugate that is weakly bound to the recognition aptamer and immobilized onto magnetic particles. The final operation (Figure 2) is based on enzymatic transduction, where DNA-invertase in the solution catalyzes the hydrolysis of sucrose into glucose and fructose, the first being measured by the PGM (Figure 3).

With such a scheme, as the concentration of the invertase conjugate released into the solution is proportional to the concentration of the target present in the sample, the PGM reader can be used to determine the target concentration [22].

Other examples where invertase or other enzymes were used in similar competitive assays are, for instance, the work of Zhang et al. [25]. In this research, the authors proposed an aptamer scheme for the detection of ochratoxin A (a fungal toxin relevant in food) and used DNAzymes to amplify the signal. The detection of the antibiotic ampicillin in milk was also presented, where a method based on biorecognition between aptamers and ampicillin conjugated onto magnetic particles was proposed [26]. Zhang et al. [27] performed a method with an aptasensor for the detection and quantification of different bacterial strains, e.g., *Staphylococcus aureus*, *Escherichia Coli*, etc. For the detection of milk adulterated with melamine, Gu et al. [28] described an aptamer sensor based on a PGM with a procedure like the previous ones. One problem with these aptamer assays with a PGM reading is related to the difference in the concentration range of the glucose and the target assays. While the former is on the 5 mM rest value, the others may need to decrease to much lower values, relying only on the activity of the enzyme.

**Figure 4:**
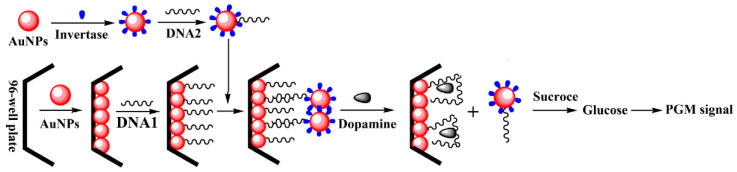
Illustrative schematic of the aptasensor for dopamine detection with PGM. Reprinted from Ref. [29], with permission of Elsevier.

Following the same detection principle, new methodologies were introduced by introducing nanotechnology components, such as gold nanoparticles, for the immobilization of the bioreceptor. Huno et al. [29] described a methodology for the detection of dopamine in serum samples by combining dopamine aptamer in a microwell plate assay. The recognition of dopamine displaces a gold nanoparticle-conjugated DNA, which carries multiple units of invertase enzyme, thus accomplishing a double amplification process if the enzyme-catalyzed hydrolysis of sucrose is accounted for (Figure 4).

Invertase was also used in sandwich format assays, where the analyte was doubly fixed by two recognition elements, with the second one labeled with the invertase enzyme. An example is the detection of myoglobin, a biomarker of cardiovascular disease, where Wang et al. [30] proposed an antibody–myoglobin–aptamer sandwich assay for the detection of this biomarker. An antibody–analyte–aptamer sandwich assay was also described by Zhu et al. [31] for the detection of vascular endothelial growth factor (VEGF), a regulator of the formation of new blood vessels from existing ones (angiogenesis); VEGF is a biomarker of several diseases such as cancer.

**Figure 5:**
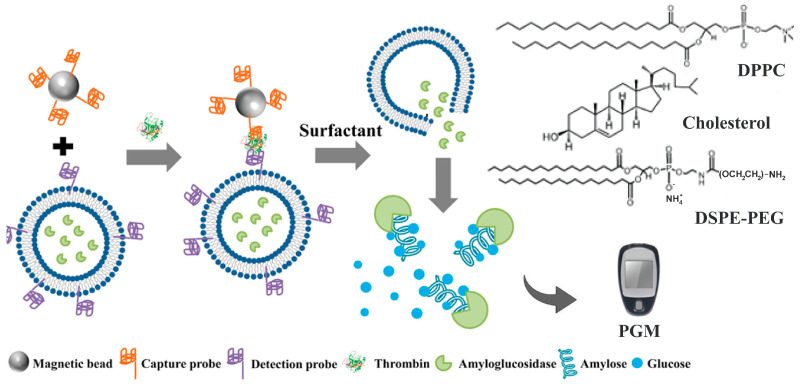
Illustrative scheme of the thrombin detection assay using encapsulated amyloglucosidase enzymes. Reprinted from Ref. [32], with permission from ACS.

**Figure 6:**
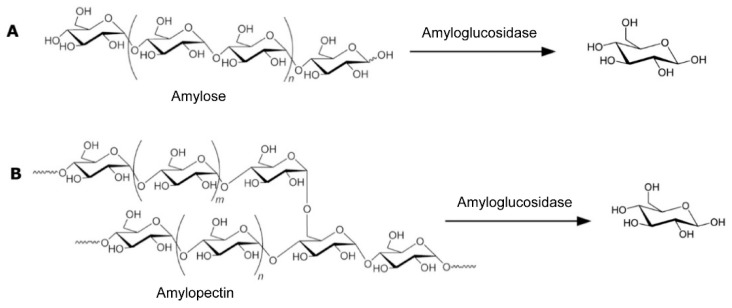
Hydrolysis of starch catalyzed by the enzyme amyloglucosidase (or glucoamylase), differentiating substrate amylose (**A**) and amylopectin (**B**). Source: own elaboration.

Lin et al. [32] presented a novel assay (Figure 5) for the detection of thrombin and C-reactive protein. In the described method, magnetic particles with immobilized capture aptamers (or antibodies) are introduced into a sample solution where the target protein (thrombin or C-reactive protein) is captured. Subsequently, liposomes with secondary detection aptamers (or antibodies) conjugated with amyloglucosidase-loaded liposomes are added. The aptamers captured the protein and aligned it in their respective secondary complexes. Upon the addition of amylose, the liposomes ruptured and released the amyloglucosidase enzyme, which catalyzed its hydrolysis (Figure 6A), yielding a large amount of glucose that could be quantitatively determined by the PGM. It is important to emphasize that this methodology is a variant of the original approach, where the enzyme used for labeling is not invertase but an alternative that also involves the appearance of glucose as a product of enzymatic hydrolysis.

Yan et al. [33] also used glucoamylase (synonym of amyloglucosidase) in an assay, and in this case, a competitive type, for the detection of ATP or cocaine based on an aptamer hydrogel modified with glucoamylase.

Table 1 summarizes this section, pointing out the representative bioassays developed using aptamers as recognition elements, enzyme labeling, and the PGM, stating the format of the assay and the enzyme transduction.

The latter cases employed intermediate enzyme encapsulation as an amplification strategy to quantify the concentration of analytes from the release of amyloglucosidase, also called glucoamylase, encapsulated in liposomes. After the recognition of the analyte, the release of the enzyme permitted the subsequent detection of produced glucose using a PGM [34]. In a similar strategy, Sun et al. [35] developed a methodology for the detection of thrombin, a protein necessary for blood coagulation. In the article, they describe a methodology based on the gold nanoclusters used as carriers for multiple glucoamylase enzymes; upon addition of amylopectin, the glucoamylase-functionalized nanoparticles catalyzed the hydrolysis of glucose (Figure 6B), generating an amplified signal in the PGM.

**Figure 7:**
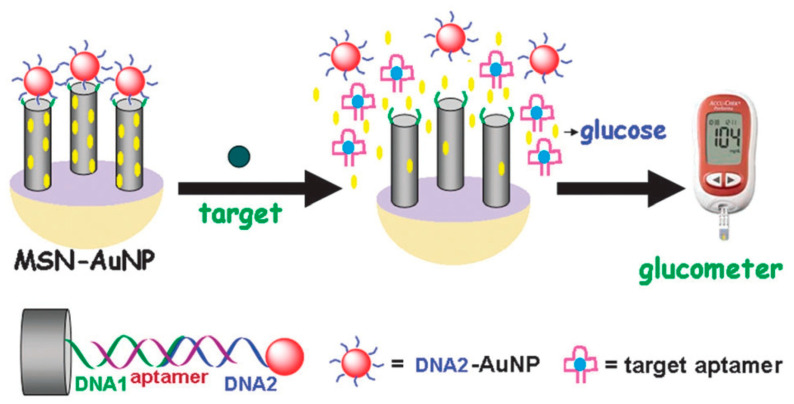
Schematic of the PGM detection methodology with mesoporous silica nanoparticles. Reprinted from Ref. [36], with permission from RSC.

In a third related variant, Tang et al. [36] designed a new ATP detection method and, in this case, used mesoporous silica nanoparticles containing entrapped glucose (Figure 7). The aptamer binds between the DNA1 captured on the silica pore and the DNA2 immobilized on gold nanoparticles. If ATP is present, the aptamer will preferentially bind to ATP. The binding of the aptamer to the analyte breaks the DNA1–aptamer–DNA2 complex and results in the release of the encapsulated glucose. Once the glucose is released into the reaction volume, it can be detected by PGM. Similar to the strategy described, Li and Tang [37] established a cocaine detection system based on the release of glucose encapsulated in titanium oxide nanotubes. Table 2 summarizes the bioassays that employ aptamers and nanocarriers to obtain the glucose signal and are next detected with the PGM.

### 2.2. Antibodies as the Recognition Element

The selectivity of the antibodies against their respective antigens lends them to be used as bioreceptors; in an immunosensor, antibodies are immobilized on the surface of the transducer, and the antigen binds by affinity to the antibody, forming an immuno-complex, that further needs some type of signal change [38]. With respect to the immunosensors using the PGM, the idea is to conjugate the antibody with a label enzyme catalyst of a reaction involving the generation or consumption of glucose.

**Figure 8:**
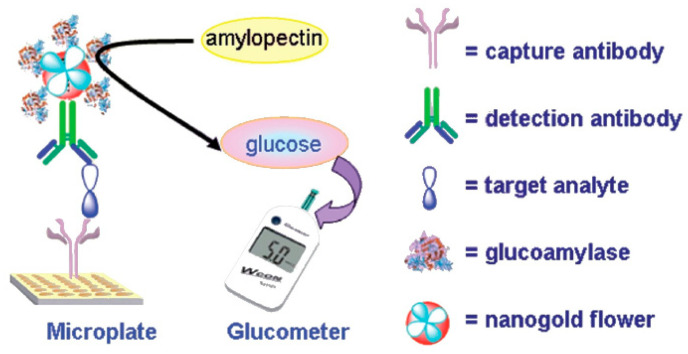
Schematic of an enzyme immunoassay for the detection of neuron-specific enolase biomarker using a PGM. Reprinted from Ref. [39], with permission from RSC.

The first example is the detection of neuron-specific enolase, a tumor marker for lung carcinoma. For this, Fu et al. [39] designed a sandwich immunoassay using glucoamylase as the labeling enzyme (Figure 8). Glucoamylase and the detection antibody were conjugated to the surface of gold nanoparticles. In the presence of neuron-specific enolase, the conjugates bind the target in a sandwich scheme, thus becoming immobilized on a microwell. Subsequently, amylopectin substrate is added, which experiences hydrolysis into glucose, catalyzed by glucoamylase and generates a signal detectable by the PGM.

Like the previous immunoassay, Wu et al. [40] reported an immunoassay for the detection of the tumor marker α-fetoprotein (AFP) by the PGM that involved a glucoamylase enzyme scheme.

Another example of the use of antibodies as bioreceptors but using invertase instead of glucoamylase as a catalyst for glucose generation is a sandwich immunoassay for the detection of Ractopamine (RAC), a drug used to adulterate animal feed because it promotes muscle mass growth. In the method used, magnetic particles were initially coated with ß-cyclodextrin. A polymer then immobilizes anti-IgG antibodies at one end and the invertase by means of gold nanoparticles. A complex is formed between the coated magnetic particles and the resulting copolymer, added to a sucrose medium, and the final glucose concentration is measured with a PGM. The RAC concentration is calculated by the linear relationship between it and the amount of glucose formed. [41].

There are also studies where glucose oxidase (GOX) was used as the signal generator. For example, Ye et al. [42] developed a method for the detection of *Cronobacter sakazakii* from powdered infant milk since it is associated with cases of meningitis in children. It is based on an immunoassay where antibodies immobilized on both GOX-coated silica nanoparticles and magnetic nanoparticles were used; a sandwich complex was formed between a first antibody immobilized on the silica nanoparticle, the *Cronobacter sakazakii*, and a second antibody immobilized on the magnetic nanoparticle. The obtained sandwich complex was separated and dispersed in a glucose solution, and the glucose concentration was determined by a PGM. A linear relationship was obtained between the decrease in glucose concentration and the logarithm of the *Cronobacter sakazakii* concentration [42]. As a summary, Table 3 presents the analyte detected, the type of assay, the enzyme used for enzyme transduction, the detection limit, and the dynamic range in articles where antibodies are used as bioreceptors.

A non-enzymatic methodology was used to detect glucose that originates encapsulated in liposomes. In this, Tang et al. [43] described a methodology using a sandwich assay for the detection of Aflatoxin B1. The analyte was captured by two antibodies: one immobilized on a microwell, and the other was bound by streptavidin to a glucose-filled nanoliposome. When a surfactant was added to the medium, the liposomes were hydrolyzed, releasing the glucose, and then the readout was performed with the PGM. Zhao et al. [34] proposed a design like the latter, with the difference that the antibody is immobilized onto iron oxide nanoparticles for the detection of phosphorylated protein P53. To detect procalcitonin, a biomarker of sepsis, Alshawawreh et al. [44] proposed a similar study. Procalcitonin was detected through an indirect sandwich immunoassay. In their scheme, there was not only the captured antibody and the detection antibody, but a third antibody was added that interacts with the detection antibody to provide more sensitivity to the assay. Table 4 summarizes the bioassays that employ antibodies and nanocarriers to encapsulate the glucose, which is next released and detected with the PGM. Even with the glucose-loaded nanocarrier, the listed examples hardly go below the pg/mL level. Again, there is a certain unmatched sensitivity between the glucose, the inherent analyte of the PGM, and the more elaborated alternatives that use liposomes.

### 2.3. Specific Peptides as Recognition Elements

The peptides considered here are short, specific amino acid sequences that have recently been proposed for use as recognition elements for biosensors with a mechanism like aptameric nucleic acids; because of this, they received the name of peptide aptamers. They are proposed as an alternative for molecular recognition, among other advantages because they can be coupled easily to electrochemical transducers [45].

**Figure 9:**
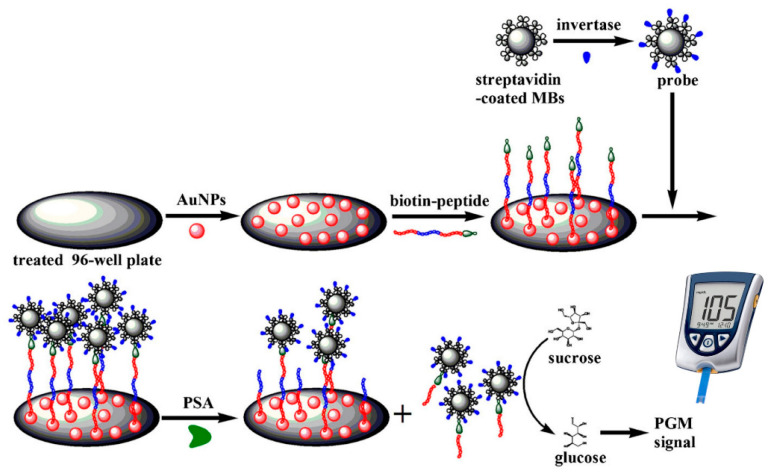
Schematic of an aptameric peptide-based sensor for PSA detection. Reprinted from Ref. [46], with permission from Springer.

For example, Luo et al. [46] proposed a biosensor based on a peptide-based competitive assay for prostate-specific antigen (PSA), an important biomarker for prostate cancer diagnosis. In the methodology (Figure 9), gold nanoparticles were added to a microwell to immobilize the biotinylated peptide. In parallel, magnetic particles were modified with streptavidin and the enzyme invertase; these were subsequently conjugated to the peptide via biotin. Once the peptide was immobilized on the microplate, if PSA was present, the peptide–biotin–invertase complex bound to magnetic particles was separated from the well plate. The peptide–biotin–invertase supernatant was then separated, and sucrose was added, which, mediated by the enzyme, was degraded into glucose. The amount of glucose detected was directly related to the concentration of PSA.

Another example of the use of peptides as bioreceptors is the design of a sensitive detection strategy for the activity of protein kinases using a PGM. Protein kinases (PKA) phosphorylate proteins, thus overseeing the regulatory functions in signal transduction pathways. Initially, PKAs phosphorylated peptides, which subsequently bound to zirconium magnetic particles; in the proposed design, the magnetic particles could selectively capture phosphorylated versus non-phosphorylated substrates. The biotinylated invertase was then added, coupling to the zirconium magnetic particles. Upon separation and addition of sucrose, the invertase catalyzed its hydrolysis, thus generating a signal for the PGM [47]. Table 5 briefly presents some characteristics of the bioassays employing peptides and the PGM.

### 2.4. DNA Genes as Recognition Elements

A DNA gene can also be used as a recognition element (gene probe) to detect its complementary nucleotide sequence (gene target) due to the specificity of the DNA hybridization process [48].

**Figure 10:**
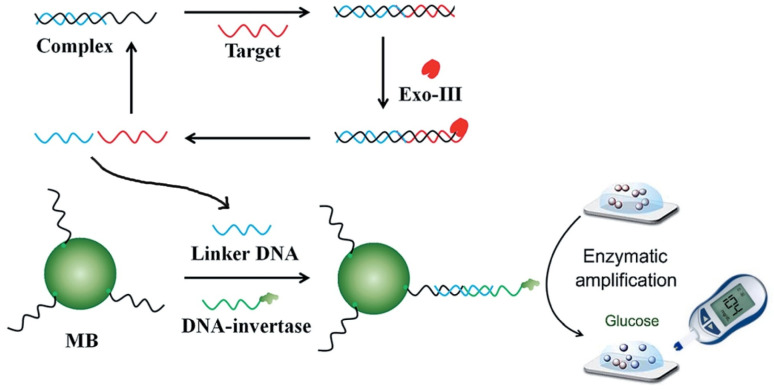
Scheme of the method for DNA detection using a PGM and exonuclease III. Reprinted from Ref. [49], with permission from RSC.

In many fields, such as clinical diagnostics, environmental analysis, etc., detecting specific DNA sequences in a simple, rapid, and sensitive manner has become increasingly important. With them, it may be possible to diagnose inherited diseases or identify pathogenic species or vegetal or animal forms. Xue-tao et al. [49] performed a DNA detection method based on signal amplification techniques assisted by exonuclease III (Exo-III) that catalyzes the cleavage of oligonucleotides on the DNA double-strand in the 3′-5′ direction without affecting the sequences. As shown in Figure 10, a DNA target–DNA-assisted complex is initially formed; if the target to be detected is present, it may hybridize together with the DNA fragment in the complex. The hybridized fragment is transferred to a system containing modified magnetic particles where the linker DNA allows the magnetic particles to bind to the DNA-invertase, hydrolyzing the sucrose so that the DNA fragment can be quantified by correlation to the measured glucose signal [49].

The same authors above, adopting a similar methodology, developed a sensor for mercury ions (Hg^2+^) using a PGM, in which DNA was immobilized on magnetic particles. DNA-invertase was captured in the presence of Hg^2+^, forming a sandwich complex; DNA-invertase catalyzes the hydrolysis of sucrose to glucose, thus finally obtaining a quantifiable signal relating the Hg^2+^ concentration to the glucose found [50].

**Figure 11:**
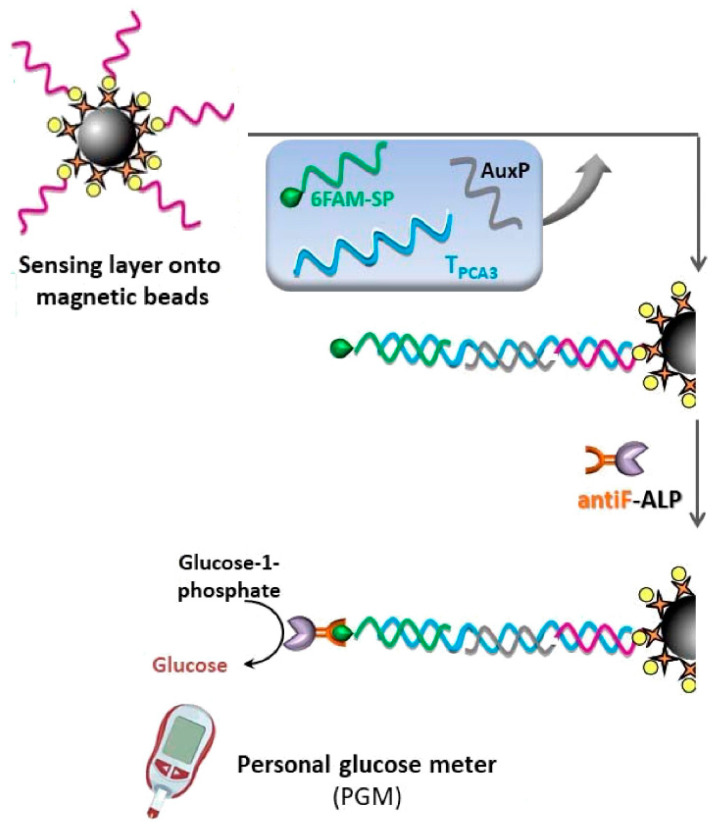
Scheme of a sandwich assay for the detection of PCA3 biomarker gene. Adapted from Ref. [51], with permission from MDPI.

In a distinct study by Arbadia et al. [51], diagnosis of prostate cancer was attempted not through the usual detection of PSA protein antigen, which suffers from low specificity, but through the PCA3 gene, a biomarker present in the urine of prostate cancer patients. In the reported study, PCA3 was detected using a magnetic particle sandwich assay, where alkaline phosphatase (ALP) was used as the signaling enzymatic reaction. The complex formed consisted of a capture probe, the PCA3 target, an auxiliary probe, and the 6FAM signal probe sequence (6FAM-SP), bound onto magnetic particles functionalized with streptavidin and biotin. For signal generation, an antibody conjugated with alkaline phosphatase (antiF-ALP) was used, to which the glucose-1-phosphate substrate was added, and the ALP enzyme catalyzed the hydrolysis of the substrate into glucose, thus generating a signal on a PGM (Figure 11).

**Figure 12:**
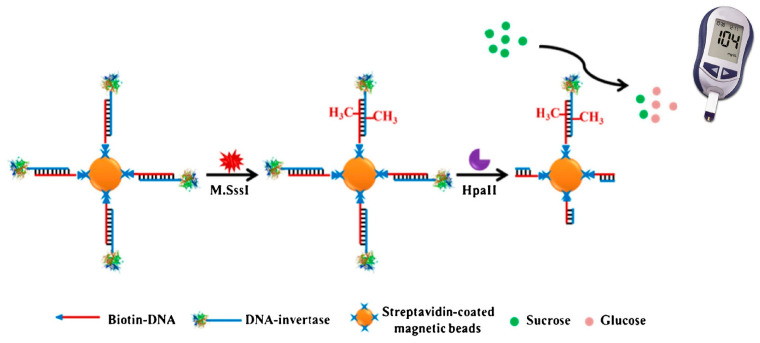
Scheme of DNA methyl transferase activity detection using a PGM. Reprinted from Ref. [52], with permission from Springer.

DNA methylation is related to several biological processes, such as the regulation of gene expression. Deng et al. [52] developed a method for the detection of DNA methyl transferase activity, where specific enzymes for DNA methylation, M.SssI MTase, and Hpa II as a restriction enzyme, were used (Figure 12). Specific DNA-invertase conjugates hybridized to the corresponding complementary DNA strands immobilized on the magnetic particles. The coding sequence served as a recognition site for the restriction enzyme Hpa II and for M.Sssi MTase. Hpa II recognizes the unmethylated DNA sequences so that the invertase will bind to the methylated sequences, where it degrades sucrose, generating a signal read by the PGM. Because the M.SssI MTase enzyme methylates first and then the restriction enzyme is added, having a high concentration of M.SssI MTase will block more active sites from Hpa II, thus releasing more invertase and generating a stronger signal in the PGM [51]. Table 6 summarizes the bioassays that employ DNA genes and PGM glucose readings to perform the determination of different analytes.

### 2.5. Other Bioreceptors

**Figure 13:**
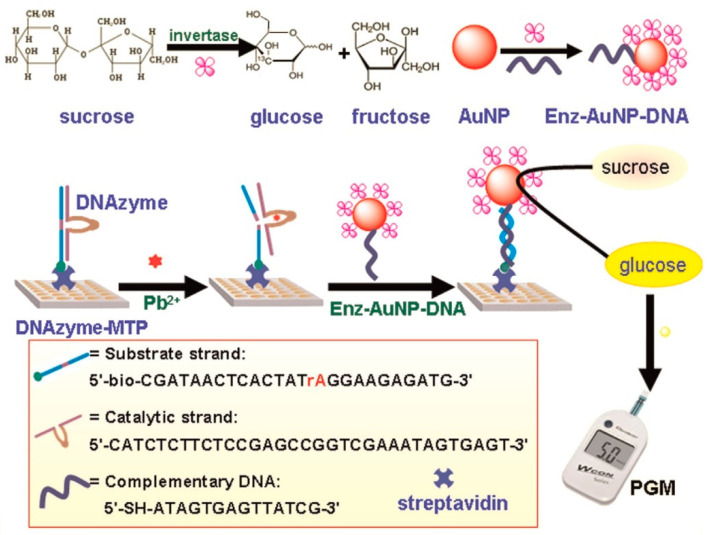
Illustrative scheme for Pb^2+^ detection by specific DNAzymes and a PGM. Reprinted from Ref. [54], with permission from Elsevier.

Deoxyriboenzymes, also called DNAzymes, are DNA molecules capable of catalyzing a specific chemical reaction, such as catalyzing the cleavage of a phosphodiester bond. Some of the DNAzymes are activated by binding to a specific analyte and are therefore used as biorecognition elements [53]. Zhang et al. [54] developed a lead ion (Pb^2+^) detection methodology (Figure 13) using a modified microplate with DNAzyme specific to Pb^2+^. Upon addition of the ions to the immobilized DNAzyme, the DNAzyme substrate chain was immobilized on the microplate and was cleaved only if lead ions were present. Previously, a complex was prepared between the invertase and gold nanoparticles and the complementary DNA fragment that hybridizes with the cleaved DNAzyme. Finally, a sucrose solution was added. The invertase catalyzed the hydrolysis of sucrose, generating glucose that could be monitored in a PGM and thus being able to relate the glucose concentration to that of the lead ion.

Liao and Li [55] reported an assay for the detection of Pb^2+^ by also using the PGM. Using a DNAzyme specific for lead ions, upon the addition of lead, the complementary DNA strand of the DNAzyme was induced to cleave. The resulting strand was hybridized with glucoamylase-DNA-gold nanoparticle conjugates, and when amylopectin was finally added, the glucoamylase present hydrolyzed it to glucose, which was subsequently detected with the PGM. To summarize the details of the usage of DNAzymes, Table 7 comments on the detected analyte, format assay, enzyme used for transduction, detection limit, and the dynamic range in the covered articles.

## 3. Other Detection Strategies: Glucose-Interfering Agents as Detected Species

The previous assays focused on the design of assays where invertase or other specific enzymes were used to convert the proper substrate into glucose for detection by a PGM. Other studies have investigated alternatives, where nanocarriers encapsulating glucose subsequently released it for detection. In both cases, glucose is oxidized by glucose oxidase (GOX)-releasing electrons that are subsequently read by a PGM. The final quantification of the analyte sought is done by correlation with the glucose concentration.

**Figure 14:**
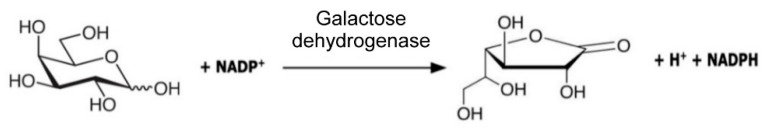
Galactose degradation reaction. Source: own elaboration.

If, instead of GOX, the sensor has galactose dehydrogenase, the personal glucose meter can detect galactose since galactose would be oxidized, generating an electrical signal that will be proportional to the galactose concentration (Figure 14). Cui et al. [56] proposed an assay based on galactose degradation for the detection of the *H1N1 influenza virus* of the influenza family.

It was recently verified that certain PGM variants can respond to nicotinamide coenzymes such as NADH or NADPH. Zhang et al. designed an assay for lactate detection based on the consumption or production of NADH by enzymatic cascade reactions catalyzed by lactate dehydrogenase and lactate oxidase [57]. The results obtained are comparable to the detection of glucose, obtaining a detection limit of 3.4 µmol/L. In connection to this, the authors combined different enzyme reactions with different inputs like metal ions, metabolites, coenzymes, and native enzymes and a PGM to obtain a biocomputing element equivalent to a logic gate, in which a diagnostic can be the result of a number of biomarker conditions [58].

**Figure 15:**
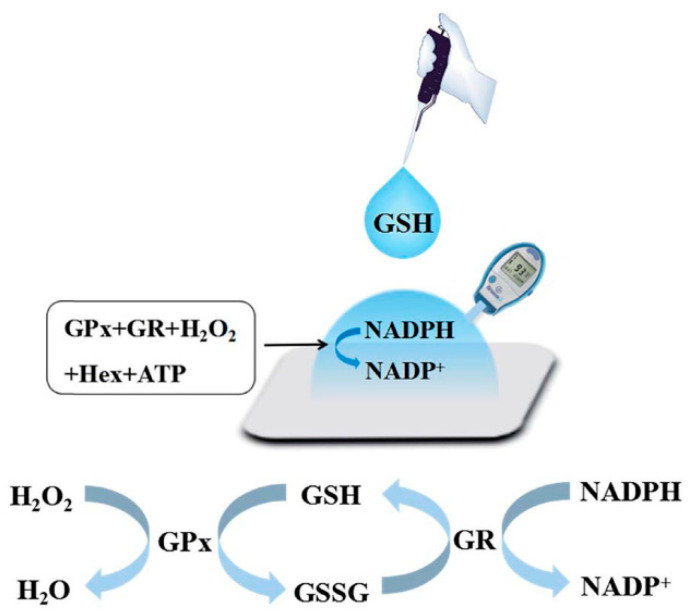
Scheme of glutathione (GSH) detection using glutathione reductase (GR) and a PGM. Other abbreviations: GSSG, glutathione disulfide; GPx, glutathione peroxidase; GR, glutathione reductase; Hex, hexokinase. Reprinted from Ref. [59], with permission from RSC.

Comparable to the above studies, Xu et al. [59] proposed an assay in which a PGM was used to detect reduced glutathione (GSH) and triglycerides in serum by means of glutathione reductase. In the assay, the PGM responded to the consumption of NADPH (Figure 15). Since NADH or NADPH are frequent coenzymes in enzymatic reactions, these studies may offer a new avenue for the detection of additional analytes.

**Figure 16:**
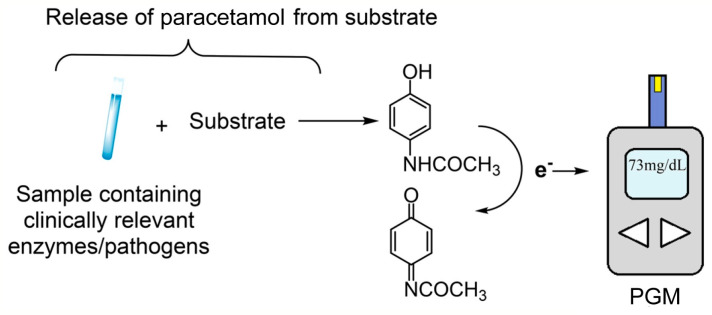
Scheme of the detection of paracetamol by a PGM. Reprinted from Ref. [60], with permission from ACS.

Lately, similar procedures have also been proposed, where the analyte resulting from the activity of the marker enzyme is an interfering substance in the PGM but, as such, provides a signal and, in the absence of glucose, can be properly calibrated. Apart from their direct determination, assays can be devised for substrates generating them as well as detection through the release of paracetamol or catechol with a PGM. Strategies have been proposed where the PGM test strip has neither glucose oxidase nor dehydrogenase present. The generation of paracetamol or catechol, departing from specific substrates and through the usage of their respective proper enzymes, may generate an electrical signal detectable by a PGM (Figure 16). This innovative strategy has been used to detect enzymes such as β-galactosidase and α-mannosidase, as well as viruses such as influenza and bacteria such as *E. coli* [60]. Table 8 summarizes cases where the PGM has been used to determine interfering substances in the electrochemical determination scheme.

## 4. COVID Applications

The recently experienced COVID-19 pandemic has highlighted the need for more rapid and accurate disease diagnostic methods at large scales and low costs, thus promoting POC diagnostic platforms. The standard diagnostic method for COVID-19 detection is the reverse transcriptase polymerase chain reaction (qRT-PCR) [61].

Despite the specificity and sensitivity of the qRT-PCR method, decentralization is needed to promote faster detection of COVID-19, a quicker diagnosis, and prevention of transmission [62]. Particularly, there are also advances in the development of POC diagnostics for COVID-19 based on a detection scheme that uses a PGM as a sensor.

**Figure 17:**
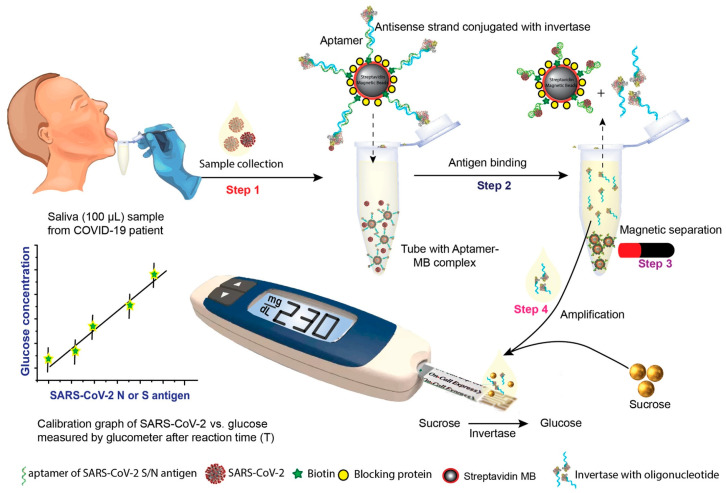
Schema of the proposed COVID-19 diagnosis based on aptamers. Reprinted from Ref. [63], with permission from Elsevier.

The assay proposed by Singh et al. [63] is based on a quantifiable antigen assay based on the use of aptamers as bioreceptors, with the glucose biosensors being capable of quantification by correlation with the glucose concentration (Figure 17). The biotin-labeled aptamer, specific for the nucleocapsid (N) or spike (S) protein of SARS-CoV-2, is conjugated by magnetic particles, and the aptamer-oligo-invertase complex is prehybridized. In the presence of viral antigens, the invertase-antigen-oligonucleotide complex can be separated. After magnetic separation, the aptamer-antibody-invertase complex is collected, and sucrose is added; the enzyme catalyzes the hydrolysis of sucrose, being the sensor reading is proportional to the viral concentration in the sample.

A second COVID-19 diagnosis strategy deserving comment is based on a system that combines the CRISPR/Cas technique with a PGM. The CRISPR/Cas technique, a recent tool for genome modification, is based on RNA-guided Cas Protein activity. Viral RNA samples are amplified by reverse transcription-assisted amplification of recombinase (RT-RAA), generating complementary DNA. Then, using the CRISPR/Cas12a technique, a specific fragment of single-stranded DNA is obtained, which is further detected by competition with a second DNA fragment of identical sequence and conjugated with invertase on magnetic particles. Upon the addition of sucrose, the presence of invertase conjugate catalyzes the hydrolysis of sucrose, producing glucose measurable by the PGM. The glucose concentration is inversely proportional to the N gene of SARS-CoV-2 [64].

Another approach combines CRISPR/Cas with a personal glucose meter where RNA from the extracted sample is amplified by isothermal amplification instead of the RT-RAA used in the previous case. The amplification products bind to Cas12a, and the protein cleaves the immobilized magnetic poly-DNA-invertase nanoparticles, releasing a series of DNA-invertase conjugates, and then catalyzes the hydrolysis of sucrose to glucose, resulting in an amplified glucose signal detectable with a PGM. The RNA concentration of COVID-19 correlates with the measured glucose signal [65]. Although, normally, these CRISPR/Cas assays in the PGM are employed for detecting DNA or RNA, this research group from Nanjing University (China) has extended this methodology to the detection of protein targets, for example, cardiac troponin I, an important biomarker for diagnosing acute myocardial infarction [66]. More recently, similar systems that rely on the CRISPR/Cas13a system have been improved for detecting small amounts of specific RNA, which has also permitted SARS-CoV-2 diagnostics. In this case, the direct interaction with the RNA virus achieves superb sensitivity (pmol·L^−1^ level) as a result of its intrinsic amplification scheme and excellent selectivity, with resolution of just one nucleotide mismatch [67].

A POC design for the virus nucleic acid detection based on a personal glucose meter was recently presented. A complex was formed between the invertase, DNA fragment 1, gene target, and DNA fragment 2. The complex was immobilized with magnetic nanoparticles, and after magnetic separation, the DNA-invertase in the presence of sucrose hydrolyzed it and thus could detect a signal in the PGM. Therefore, the amount of invertase captured by the magnetic particles is directly related to the target gene concentration [68].

## 5. Conclusions

In conclusion, personal glucose meters can be successful POC devices because of their sensitivity, selectivity, portability, and cost savings; moreover, they are instruments by themselves; hence, they do not need any other expensive or complicated instrumentation. They can have an important clinical utility for the quantification of a wide range of analytes, thus favoring mass screening in health care or speeding up diagnoses.

This paper presented a brief review of the extension of the personal glucose meter to analytes other than glucose in POC environments. With this idea, the PGM can be extended to additional branches of biomedical analysis, such as rapid diagnosis of COVID-19 or biomarkers relevant to prostate cancer, among others. It also has applications for food and environmental analyses.

For practical application, a number of issues related to the current PGM system will have to be solved. First, PGMs are designed to measure glucose in the ranges from 30 to 600 mg/dl, depending on the sensor used [19], which covers the dynamic range of glucose in people with diabetes. Nevertheless, there is a challenge when it comes to measuring other analytes, most of which are also present in low concentrations on a physiological scale. Therefore, for the proper application in the analysis of protein biomarkers or genes, instruments permitting the achievement of lower limits of detection are needed. Alternatively, the use of amplification strategies to achieve the same outcome could be mentioned. Secondly, since glucose is usually present in physiological samples in various amounts, detection methodologies should take endogenous glucose into account. Finally, for it to be useful as a POC test for other analytes, it must be easy to use. The recognition mechanisms of the sensors are still being developed, prioritizing simple tests where the user with a minimal amount of sample (blood, sweat, urine, etc.) in a single step would obtain a result. No such simplified system has yet been reported in the literature, but as the remaining sensor challenges are solved, it is possible that PGMs will be implemented as POC tests for the detection of analytes other than glucose, opening exciting possibilities. Of course, the dream of a device providing a quick analysis of a given biomarker with the unique manual operation of placing a portion of blood or urine sample in the device is still far from being accomplished; probably, integration with microfluidics and/or lab-on-a-chip technologies would be some possibilities for solving such challenges.

## Figures and Tables

**Table 1:** Summarizing table of bioassays developed using aptamers as the recognition element and the PGM.

Analyte	Assay Type	Enzyme	Detection Limit (LOD)	Dynamic Range	Reference
Cocaine	Competitive	Invertase	3.4 mmol/L	0–500 mmol/L	[22]
Adenosine	Competitive	Invertase	18 mmol/L	0–1000 mmol/L	[22]
Interferon-γ	Competitive	Invertase	2.6 μmol/L	0–400 mmol/L	[22]
Uranium	Competitive	Invertase	9.1 nmol/L	0–200 mmol/L	[22]
Ocratoxin A	Competitive	Invertase	88 pg/L	0.001–300 ng/mL	[25]
Ampicillin	Competitive	Invertase	25 nmol/L	2.5 × 10^−10^–1.2 × 10^−7^ mol/L	[26]
*Staphyllococcus aureus*	Competitive	Invertase	1.0 × 10^5^ CFU/mL	1.2 × 10^5^–1.2 × 10^8^ CFU/mL	[27]
*E. coli*	Competitive	Invertase	1.0 × 10^6^ CFU/mL	-	[27]
Melamine	Competitive	Invertase	0.33 mmol/L	-	[28]
Dopamine	Competitive	Invertase	3 μmol/L	0.08–100 mmol/L	[29]
Myoglobin	Sandwich	Invertase	50 pmol/L	2.2–27.8 mmol/L	[30]
VEGF	Sandwich	Invertase	1.2 ng/L	3–100 ng/L	[31]
Thrombin	Sandwich	Glucoamylase	1.7 nmol/L	0–100 nmol/L	[32]
Cocaine	Competitive	Glucoamylase	3.8 μmol/L	3.8–750 μmol/L	[33]
ATP	Competitive	Glucoamylase	-	0.0–1.0 mmol/L	[33]

**Table 2:** Detected analyte summary table, type of test, nanocarriers used for transduction, detection limit, and dynamic range.

Analyte	Assay Type	Nanocarriers	Detection Limit (LOD)	Dynamic Range	Reference
Thrombin	Sandwich	Liposome	1.7 nmol/L	0–100 nmol/L	[32]
Cocaine	Competitive	Hydrogel	3.8 μmol/L	3.8–750 μmol/L	[33]
ATP	Competitive	Hydrogel	-	0.0–1.0 mmol/L	[33]
Thrombin	Sandwich	Gold nanocluster	0.001 nmol/L	0.05–100 nmol/L	[35]
ATP	Competitive	Mesoporous silica	8.0 μmol/L	8.0–800 μmol/L	[36]
Cocaine	Competitive	Nanotubes TiO_2_	5.2 nmol/L	10–600 nmol/L	[37]

**Table 3:** Summary of the bioassays developed using antibodies as the bioreceptor and final detection of glucose employing the PGM.

Analyte	Assay Type	Enzyme	Detection Limit (LOD)	Dynamic Range	Reference
Neuron-specific enolase	Sandwich	Glucoamylase	8 pg/mL	0.01–30 ng/mL	[39]
α-fetoprotein	Sandwich	Glucoamylase	0.03 ng/mL	0.1–50 ng/mL	[40]
Ractopamine	Sandwich	Invertase	0.34 mg/kg	1–40 ng/mL	[41]
*Cronobacter sakazakii*	Sandwich	Glucose oxidase	42 CFU/mL	9.0 × 10^2^–9.0 × 10^7^ CFU/mL	[42]

**Table 4:** Table summarizing the analyte detected and the type of assay in examples that use antibodies and nanocarriers to achieve glucose transduction.

Analyte	Assay Type	Nanocarriers	Detection Limit (LOD)	Dynamic Range	Reference
Aflatoxin B1	Sandwich	Liposome	0.6 pg/mL	0.001–10 ng/mL	[43]
Phospho-P53	Sandwich	Liposome	50 pg/mL	0.3–20 ng/mL	[34]
Procalcitonin	Sandwich	Liposome	0.15 nmol/L	0.15–15.4 nmol/L	[44]

**Table 5:** Detected analyte summary table, type of test, nanocarriers used for transduction, detection limit, and dynamic range in articles where peptides are used as bioreceptors.

Analyte	Assay Type	Enzyme	Detection Limit (LOD)	Dynamic Range	Reference
PSA	Competitive	Invertase	30 pg/mL	0.08–7 ng/mL	[46]
Protein kinase	Sandwich	Invertase	0.1 U/µL	0.0001–0.001 U/µL	[47]

**Table 6:** Detected analyte summary table, type of test, detection limit, and dynamic range in articles where DNA genes are used as bioreceptors.

Analyte	Assay Type	Enzyme	Detection Limit (LOD)	Dynamic Range	Reference
DNA gene	Sandwich	Invertase	0.5 pmol/L	0.5–100 pmol/L	[49]
Mercury (Hg^2+)^	Sandwich	Invertase	8.0 nmol/L	8.0–1000 nmol/L	[50]
PCA3 gene	Sandwich	Alkaline Phosphatase	2.5 pmol/L	5–100 pmol/L	[51]
M.SssI MTase Activity	Sandwich	Invertase	0.37 U/mL	0.5–80 U/mL	[52]

**Table 7:** Summary of two examples that employ DNAzymes and the PGM for detecting activators of the enzyme activity.

Analyte	Assay Type	Enzyme	Detection Limit (LOD)	Dynamic Range	Reference
Pb^2+^	Competitive	Invertase	1.0 pmol/L	1.0–800 pmol/L	[54]
Pb^2+^	Competitive	Glucoamylase	2.0 pmol/L	5.0–500 pmol/L	[55]

**Table 8:** Summary table with other detection strategies in which interfering agents are determined with the PGM, listing the detection method, detection limit, and dynamic range.

Analyte	Method	Detection Limit (LOD)	Dynamic Range	Reference
*Influenza virus H1N1*	Directly measured galactose generation with PGM	100 pfu	–	[56]
L-lactate	Measured NADH consumption or production with PGMs	0.034 mM	0.05–2.0 mM	[57]
Glutathione	Measured NADH consumption or production with PGMs	0.8 mM	2.0–15.0 mM	[59]
Triglycerides	Measured NADH consumption or production with PGMs	0.17 mM	0.8–15.0 mM	[59]
β-galactosidase	Specific enzymes generate paracetamol or catechol and are measured directly with the PGM	0.01 U	–	[60]
α-mannosidase	Specific enzymes generate paracetamol or catechol and are measured directly with the PGM	0.01 U	–	[60]
*E. coli*	Specific enzymes generate paracetamol or catechol and are measured directly with the PGM	100 CFU/mL	–	[60]
*Influenza virus H1N1*	Specific enzymes generate paracetamol or catechol and are measured directly with the PGM	1 PFU	–	[60]

## Data Availability

Data sharing is not applicable.
